# Classify Hyperdiploidy Status of Multiple Myeloma Patients Using Gene Expression Profiles

**DOI:** 10.1371/journal.pone.0058809

**Published:** 2013-03-15

**Authors:** Yingxiang Li, Xujun Wang, Haiyang Zheng, Chengyang Wang, Stéphane Minvielle, Florence Magrangeas, Hervé Avet-Loiseau, Parantu K. Shah, Yong Zhang, Nikhil C. Munshi, Cheng Li

**Affiliations:** 1 Department of Bioinformatics, School of Life Science and Technology, Tongji University, Shanghai, China; 2 Inserm UMR892, CNRS 6299, Université de Nantes, Centre Hospitalier Universitaire de Nantes, Unité Mixte de Genomique du Cancer, Nantes, France; 3 Department of Biostatistics and Computational Biology, Dana-Farber Cancer Institute and Harvard School of Public Health, Boston, Massachusetts, United States of America; 4 Department of Medical Oncology, Dana-Farber Cancer Institute and Harvard Medical School, and Boston VA Healthcare System, Boston, Massachusetts, United States of America; Queen's University Belfast, United Kingdom

## Abstract

Multiple myeloma (MM) is a cancer of antibody-making plasma cells. It frequently harbors alterations in DNA and chromosome copy numbers, and can be divided into two major subtypes, hyperdiploid (HMM) and non-hyperdiploid multiple myeloma (NHMM). The two subtypes have different survival prognosis, possibly due to different but converging paths to oncogenesis. Existing methods for identifying the two subtypes are fluorescence in situ hybridization (FISH) and copy number microarrays, with increased cost and sample requirements. We hypothesize that chromosome alterations have their imprint in gene expression through dosage effect. Using five MM expression datasets that have HMM status measured by FISH and copy number microarrays, we have developed and validated a K-nearest-neighbor method to classify MM into HMM and NHMM based on gene expression profiles. Classification accuracy for test datasets ranges from 0.83 to 0.88. This classification will enable researchers to study differences and commonalities of the two MM subtypes in disease biology and prognosis using expression datasets without need for additional subtype measurements. Our study also supports the advantages of using cancer specific characteristics in feature design and pooling multiple rounds of classification results to improve accuracy. We provide R source code and processed datasets at www.ChengLiLab.org/software.

## Introduction

Multiple myeloma (MM) is a plasma cell malignancy characterized by complex and heterogeneous cytogenetic abnormalities [1]. In the U.S., multiple myeloma is the second most common hematological malignancy and constitutes 1% of all cancers [2]. Aneuploidy, defined as copy number changes of chromosomes or regions, is a common feature of many human cancers including MM [3]. Common chromosome amplification and deletion events can be used as a feature to subdivide multiple myeloma into two subtypes. Using fluorescence in situ hybridization (FISH), multiple myeloma is divided into hyperdiploid multiple myeloma (HMM) and non-hyperdiploid multiple myeloma (NHMM) [4], [5]. Approximately 55–60% of patients have the hyperdiploid karyotype, with trisomies of eight specific chromosomes, including chromosome 3, 5, 7, 9, 11, 15, 19 and 21 [6], [7]. The remaining cases form the non-hyperdiploid group, frequently involving translocations and hemizygous deletion of chromosome 13.

The two MM subtypes have different prognosis and survival outcome. Patients with hyperdiploid multiple myeloma tend to have a better prognosis than those with non-hyperdiploid subtype [4], [8], [9]. Therefore, cytogenetic and genomics based prognosis models should use the subtype status as a covariate to help uncover novel and independent factors for prognosis and drug response. Furthermore, the two MM subtypes may be driven by different but overlapping paths to oncogenesis. As genomics profiling of RNA and DNA is increasingly used in myeloma studies, with the subtype status of patient samples we can perform meta-analysis of genomics profiling datasets to study common and specific pathways dysregulated in the two subtypes and corresponding drug candidates.

However, most genomics profiling myeloma studies do not have HMM status measured. The existing standard method for distinguishing the two subtypes is FISH, which measures a few chromosomes' trisomy status and can be inaccurate for determining the hyperdiploidy status of cancer samples. Using array comparative genomics hybridization (aCGH) or SNP arrays to detect genome-wide copy number changes can also readily identify the two major subtypes [10], but paired copy number and expression datasets are few. In this study, we hypothesize that chromosome alterations have their imprint in gene expression through dosage effect, and aim to build a robust, cross-platform method to classify multiple myeloma subtypes using gene expression profiles. The resulting MM subtype status will greatly help the analysis of existing and future myeloma profiling datasets in better understanding the commonalities and differences of disease biology and prognosis in HMM and NHMM subtypes.

## Materials and Methods

### Gene expression profiles

GSE6477, GSE19784, GSE6401 are 3 gene expression profiling datasets from GEO (http://www.ncbi.nlm.nih.gov/geo/) [11]–[13]. All of them have been separated into HMM and NHMM with FISH, and the exact method are in the corresponding papers. Among these data, GSE6477 and GSE6401 are of the same array platform (HG-U133A) while GSE19784 is different (HG-U133_Plus2). To test classification accuracy using copy number-based HMM status, we use GSE29023 with paired expression and array CGH profiling [14], and GSE12896 and GSE39754 with paired expression and SNP array profiling [10].

### Training, validation and test datasets

We use GSE6477 and GSE19784 as training data and validation data to build the method and select parameters, and use GSE6401 as test data to test the accuracy of method. GSE6477 was one of the early datasets about multiple myeloma with identified subtype by FISH. It contains 140 samples with the same number of two subtypes (70 HMM and 70 NHMM). We use GSE19784 to improve the method for cross platform/dataset classification and as validation data to minimize over-fitting. The rest datasets are used for testing the classification accuracy.

### Microarray data processing

All gene expression profile analysis procedures were performed using R (version 2.13.1). Package ‘affy’ was used for primary process [15]. Custom CDF files were used for R 2.13 (http://brainarray.mbni.med.umich.edu/). The probe level data was converted to expression values using the BioConductor project with the Robust Multi-Array average (RMA) procedure, in which perfect match intensities were background adjusted, quantile-quantile normalized, and log2-transformed [16], [17]. R package ‘kknn’ was used for KNN and KKNN classification (http://cran.r-project.org/web/packages/kknn/).

### Definition of trisomy chromosomes

Based on frequent trisomy status of eight chromosomes in HMM, we separate genes into two groups: trisomy-chromosome genes (genes on chromosomes 3, 5, 7, 9, 11, 15, 19 and 21) and nontrisomy-chromosome genes (genes on the rest chromosomes) [6]. We use UCSC hg19 RefSeq gene table as reference (http://genome.ucsc.edu/).

### Determine HMM status from copy number microarray data

We used two test datasets with paired copy number microarray samples as gold standard for HMM status. In GSE29023, for each sample we computed the ratio between the median copy number of the trisomy chromosomes and that of the non-trisomy chromosomes, and plotted the histogram of the ratios. We expected this ratio to be around 1.5 (3 copy/2 copy) for HMM samples and 1 (2 copy/2 copy) for NHMM samples, since HMM samples usually have 3 copies in most of the 8 trisomy chromosomes, but NHMM samples have 2 copies in these chromosomes. With the expectation that the patients should be separated into two groups, we observed the histogram and chose the boundary 1.125 as half-way between the two peaks in the distribution. The fact that we didn't observe a cutoff midway between 1 and 1.5 could be due to sample normalization during microarray analysis, where the overall signal distribution in one sample is adjusted to be similar across all samples. This may lead to a downward shifting of all signals in a HMM sample, where 8 chromosomes are usually in trisomy. A cutoff of 1.10 or 1.15 is also possible from this histogram, but this leads to only 3 samples to be assigned differently and a difference in accuracy of <2.6% (3/115 of samples in GSE29023).

In GSE12896, we plotted the distribution of the median copy number of the trisomy chromosomes and expected the median to be around 3 for HMM and around 2 for NHMM. We observed the corresponding two peaks, although the HMM samples peak at 2.6 due to possible normalization effect as above. We chose the midway value (2.3) between the two peaks as the cutoff to call HMM and NHMM samples from the copy number data.

## Results

### Develop classification method for HMM status by cross-validation

**Figure 1:**
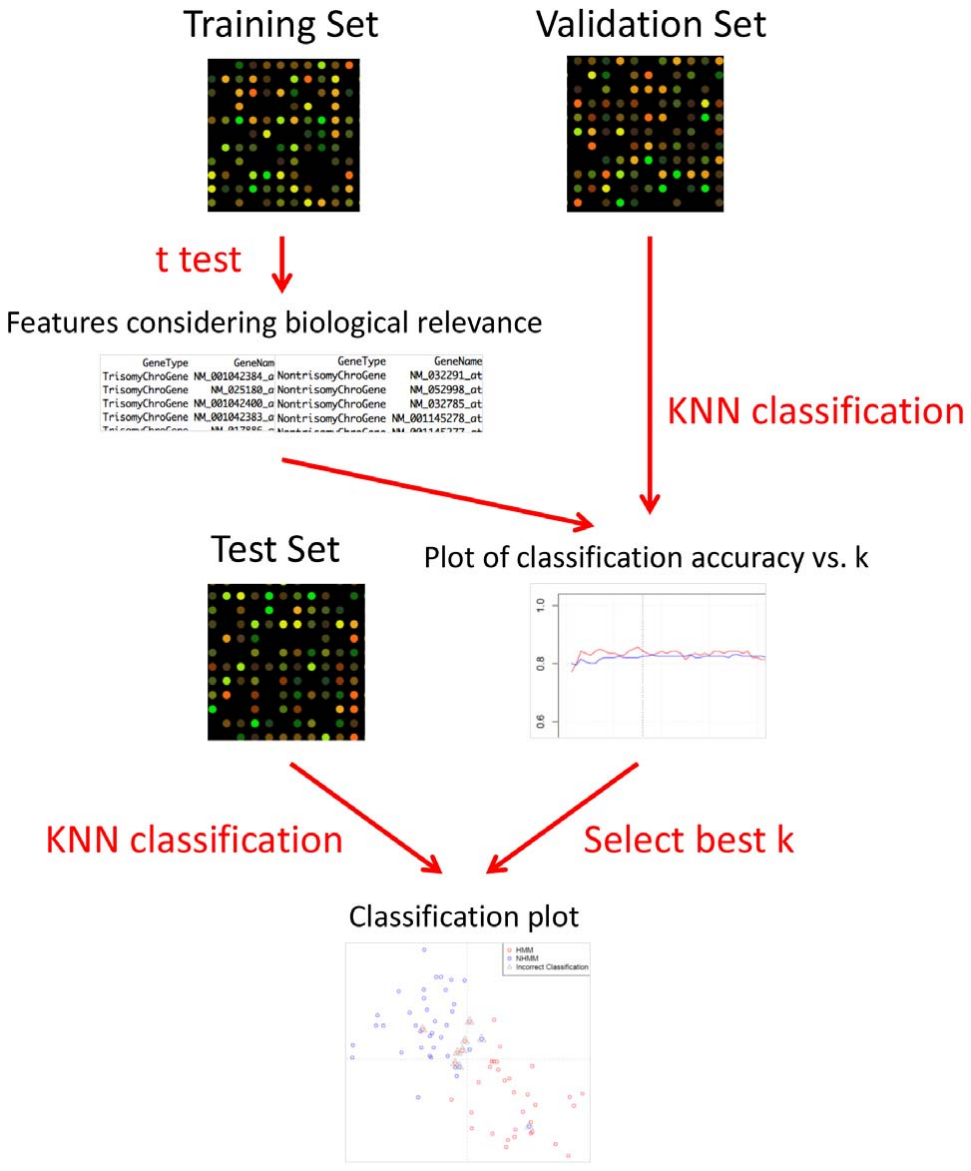
A general flowchart of the classification analysis steps.

We used K-nearest neighbor (KNN) method and leave-one-out cross-validation (LOOCV) to develop the classification method for HMM status. Based on this method, we leave one sample out of GSE6477 and use the remaining data as training samples for fitting the classification model. A list of genes differentially expressed between HMM and NHMM of the training samples are used to construct a two-dimensional feature vector to classify the left-out sample. The overall classification accuracy from cross-validation is used to select the optimal model parameters. An overview of the procedure is in Figure 1 and the detailed steps are as follows.

First, using GES6477, we left one sample out and used the rest 139 samples to perform Student's t-test between HMM and NHMM using each gene's expression data. Controlling for FDR (False Discovery Rate) that is less than 0.01, we obtained a list of genes that are differentially expressed between HMM and NHMM. We subdivided these genes into trisomy-chromosome genes (TC genes) and nontrisomy-chromosome genes (NTC genes) according whether a gene belongs to trisomy chromosomes or nontrisomy chromosomes (see Methods). Table S1 lists the intersection genes used in the 140 leave-one-out classification models.

**Figure 2:**
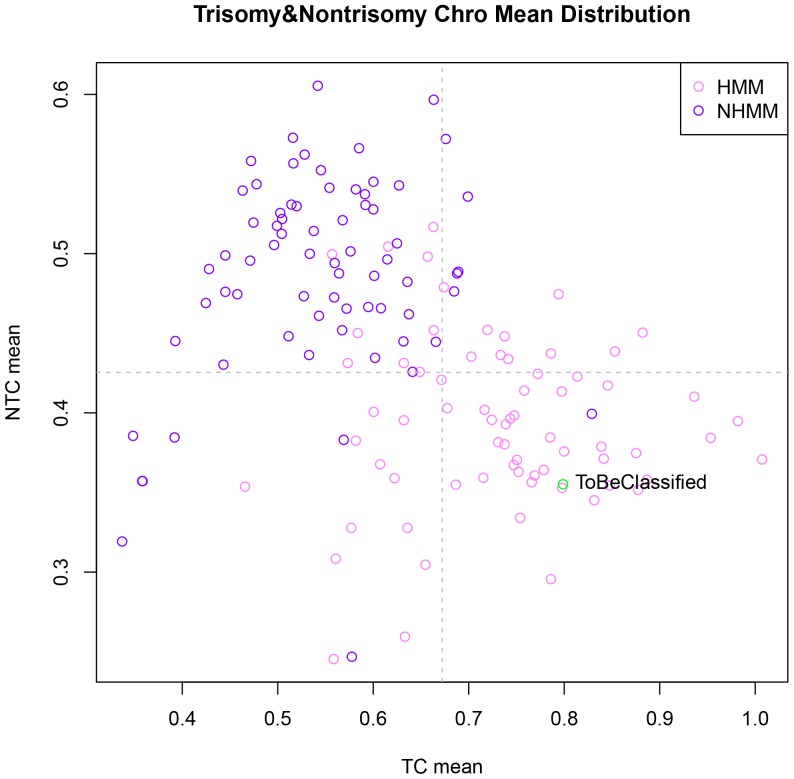
Scatterplot of trisomy and nontrisomy chromosome means of GSE6477 samples. Each point represents a patient sample. ‘TC mean’ on X-axis is the average scaled expression values for the genes on the 8 trisomy chromosomes. ‘NTC mean’ on Y-axis is the average scaled expression values for the genes on nontrisomy chromosomes. The light purple circles are HMM samples and the dark purple circles are NHMM samples determined by FISH. The green circle is the left-out sample to be classified.

Next, we standardized GSE6477 to make the mean of each sample's expression values of all genes to be 0 and the variance of each sample to be 1, and got the GSE6477_scale data. This step is necessary for cross-platform/dataset classification in the next section. Then, for each sample in the training and validation sets of GSE6477_scale, we computed the mean expression of the TC genes and NTC genes, respectively, and denote the two means as TC mean and NTC mean. Figure 2 shows that when plotting NTC mean vs. TC mean for all the samples, HMM and NHMM samples largely separate.

**Figure 3:**
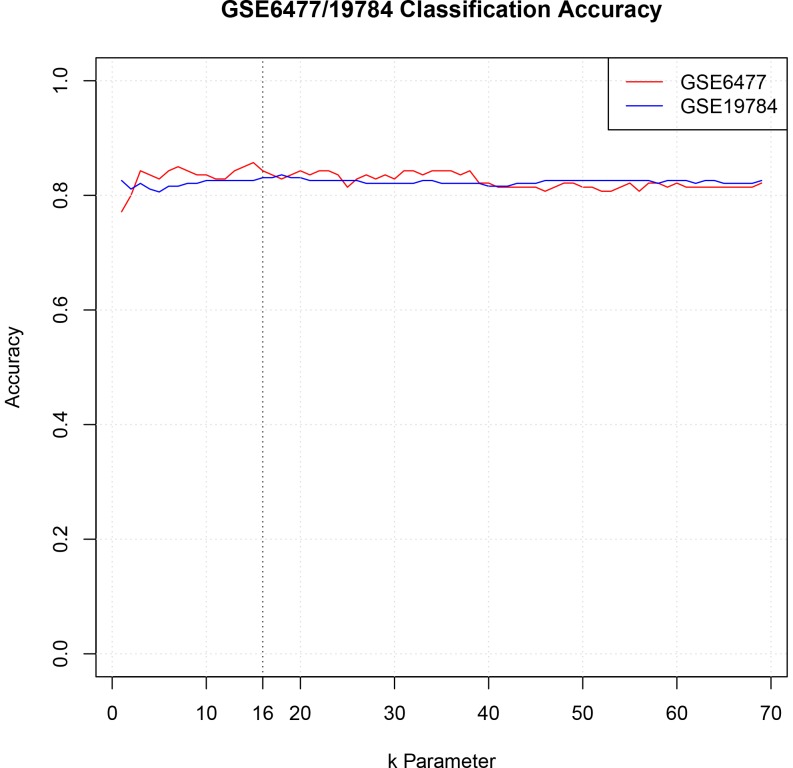
GSE6477/19784 classification accuracy for different k values in KNN. When k equals 16, both datasets have good classification accuracy.

Last, we used TC mean and NTC means as the feature values of each sample to perform K-nearest neighbor algorithm (KNN) to classify the left-out sample using the 139 training samples. KNN is a method for classifying objects based on closest training examples in the feature space, which is our core algorithm for classification [18], [19]. The main parameter of KNN is the k value, the number of neighbors considered [18]. We used different k parameters from 1 to 69 to explore the best k value for classification. For a given k value, we performed leave-one-out cross-validation to classify every sample, and compared the classified subtypes to the original FISH-based subtypes of all samples to assess the overall classification accuracy. Figure 3 shows the classification accuracy with various value of k from 1 to 69 for GSE6477 cross-validation results. When the k value is more than 2, the accuracy of classification is more than 80%.

### Develop cross-dataset classification method

**Figure 4:**
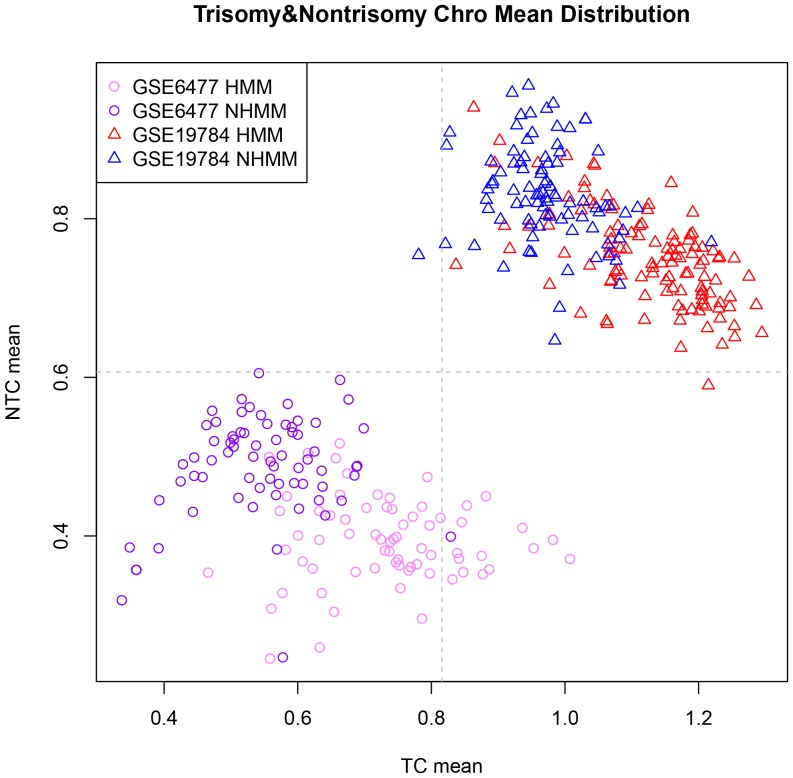
Scatterplot of trisomy and nontrisomy chromosome means of both GSE6477 and GSE19784 samples. See Figure 2 legend for details. The top right part are GSE19784 samples separated into two FISH subtypes by color. The two datasets are far from each other.

We next used GSE19784 to check the applicability of the classification method developed from GSE6477 when applied to an independent dataset and patient cohort. GSE19784 uses a different array platform, comes from a different research study and has more MM samples (201 MM samples with FISH status). These factors may affect the accuracy of cross-dataset classification. To minimize platform and batch differences between the two datasets, we also standardized GSE19784 to make the mean expression values of all genes in each sample to be 0 and the variance of each sample to be 1, and obtained the GSE19784_scale data. To apply a classification model from one cross-validation run of GSE6477 (Figure 2), we retrieved the corresponding differential expressed gene list from the classification model and computed TC mean and NTC mean for every sample of GSE19784_scale. However, the distribution of the two data sets was far in distance, making the KNN classification challenging and less accurate across datasets (Figure 4).

**Figure 5:**
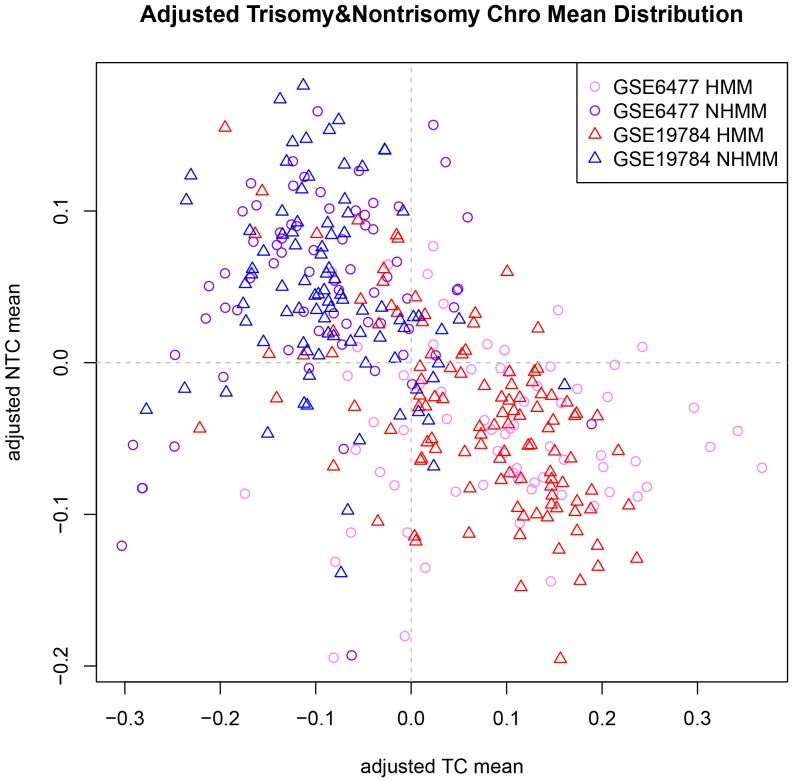
Scatterplot of adjusted trisomy and nontrisomy chromosome means of both GSE6477 and GSE19784 samples. Adjusting and overlapping the distributions of GSE6477 and GSE19784 makes classification more accurate. For all the sample points in a dataset from Figure 4, we shift their locations by the same amount, so the center of the samples points locates at (0, 0).

On a closer look, we observed that the relative locations and overall shape of HMM and NHMM samples within a dataset are similar for the two datasets (Figure 4). In order to adjust for dataset specific characteristics, we overlaid the two datasets' distributions (Figure 5). Specifically, we shifted the locations of all the sample points in a dataset in the TC mean vs. NTC mean plot, so that all the sample points in the dataset have the 0 average in both X-axis and Y-axis values. As the result, both datasets have their data points centered at (0, 0) and their overall distributions are overlapping (Figure 5), facilitating KNN classification across array platform and datasets.

Last, we used the adjusted TC mean and NTC mean as sample features to classify samples in GSE19784_scale using models trained from GSE6477. We classified each sample in GSE19784 140 times using each of the leave-one-out cross-validation models from GSE6477. The subtype classified as the majority of 140 times is the final classified subtype of the sample. We then compared the classified subtype of GSE19784 samples to their FISH-based subtype to check the cross-dataset classification accuracy. Figure 3 shows that for k>8, we obtained classification accuracy >82%.

### Test the classification method using independent datasets with FISH and copy number-based HMM status

**Figure 6:**
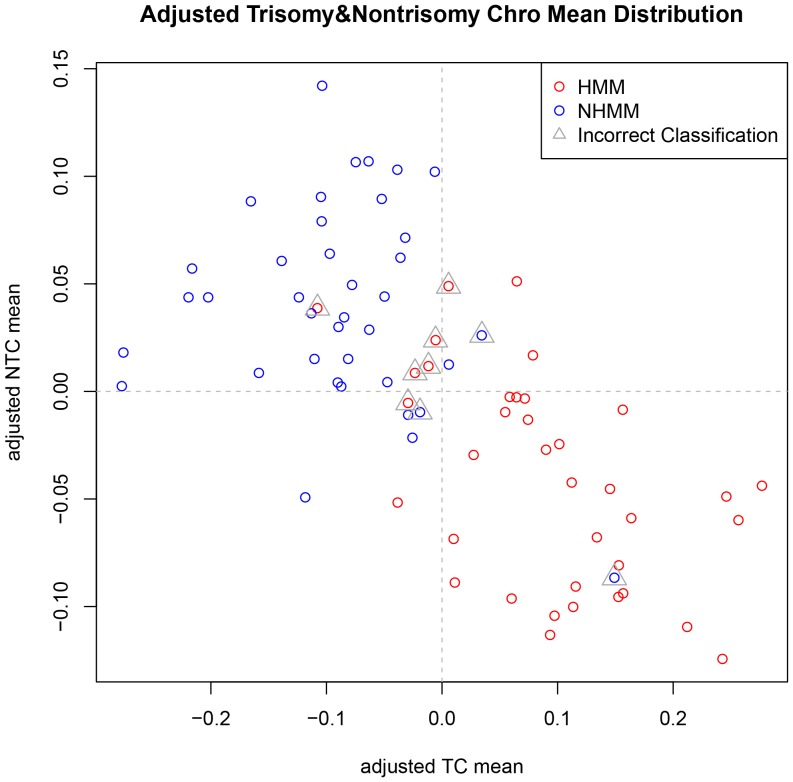
Scatterplot of adjusted trisomy and nontrisomy chromosome means of GSE6401. The green triangles indicate misclassified samples. Most of them locate at the boundary area between the samples of two FISH subtypes.

Using the classification results of GSE6477 (within-dataset cross-validation) and GSE19784 (cross-dataset classification), we explored values of k from 1 to 69 that resulted in high classification accuracy (Figure 3). We selected k = 16 to classify three independent test datasets, GSE6401, GSE29023 and GSE39754. We performed similar procedure as above, using the GSE6477-trained models to classify GSE6401, and obtained a cross-dataset classification accuracy of 0.88 when compared to FISH (Figure 6).

**Figure 7:**
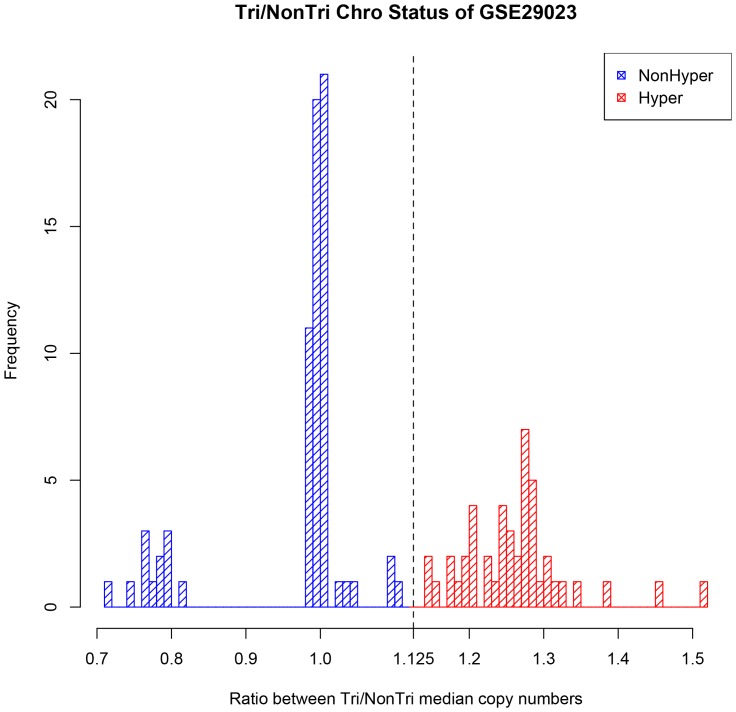
Determine the copy number-based HMM status in GSE29023. For each sample, **t**he ratio between the median array-CGH based copy numbers of the trisomy chromosomes and that of the non-trisomy chromosomes is computed. See Methods for details. With the expectation that the patients should be separated into two groups, we observed the histogram and chose the boundary 1.125 as half-way between the two peaks in the distribution.

We also tested the classification method using two MM expression datasets with paired copy-number profiling samples. The first is GSE29023, consisting of paired gene expression and array CGH samples [14]. Array CGH is a technique to detect genomic copy number variations at a high resolution. We first utilized the CGH data to compute the ratio between the median copy number of trisomy chromosomes and of nontrisomy chromosomes for each patient. For HMM samples we expect higher ratios because they tend have three copies in the trisomy chromosomes. The histogram of the ratios shows a division of samples (Figure 7), and we set the cutoff as 1.125 to call real HMM status based on copy numbers. Last, we applied the expression-based classification method to call HMM status, which agreed with the copy number-based HMM status at a accuracy of 0.835 (96/115).

**Figure 8:**
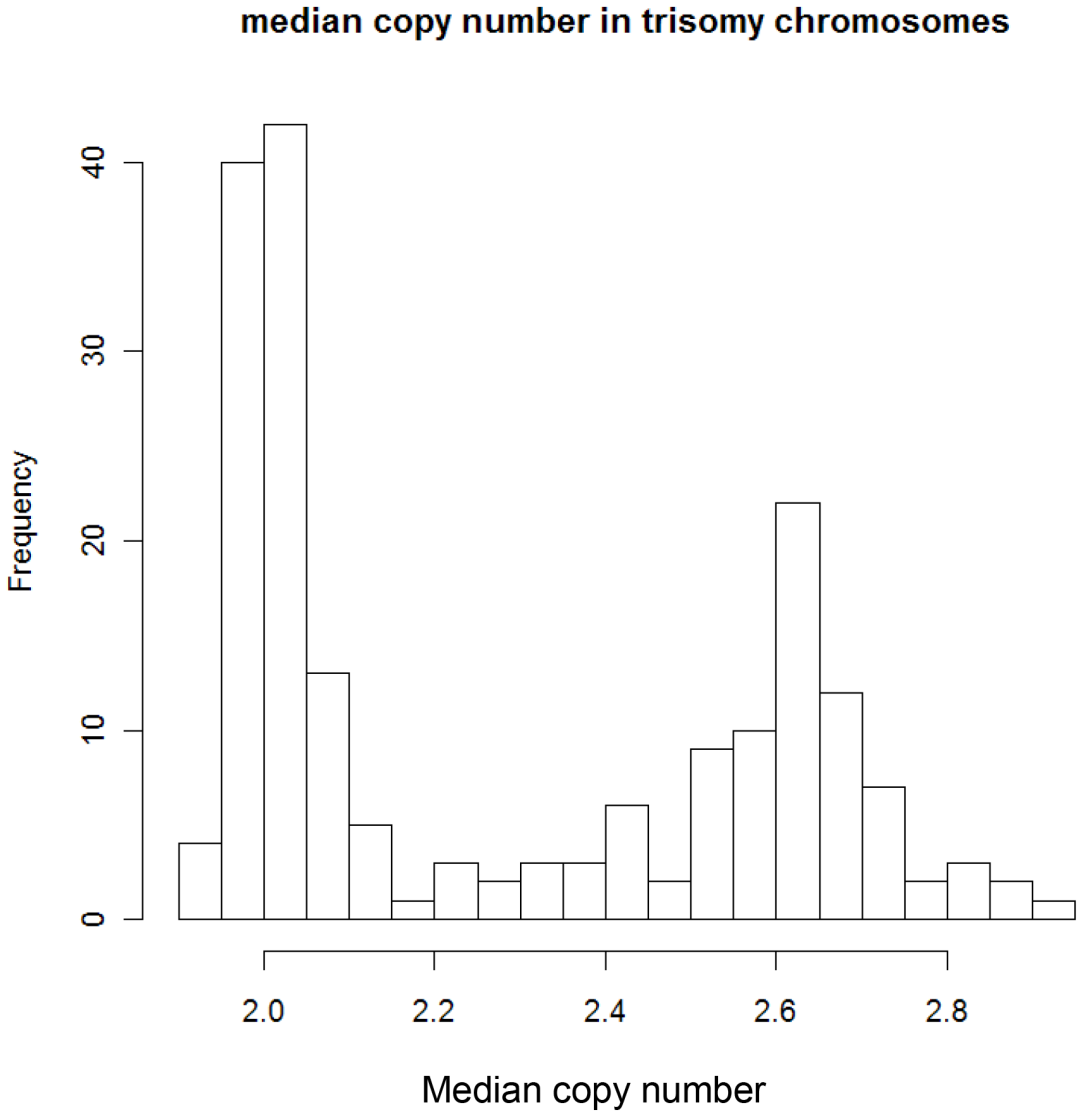
Determine the copy number-based HMM status in GSE12896. The median copy number of SNPs in trisomy chromosomes is computed for each sample. The histogram suggests a cutoff of 2.3 to determine HMM status (median copy >2.3 for HMM samples). See Methods for details.

**Figure 9:**
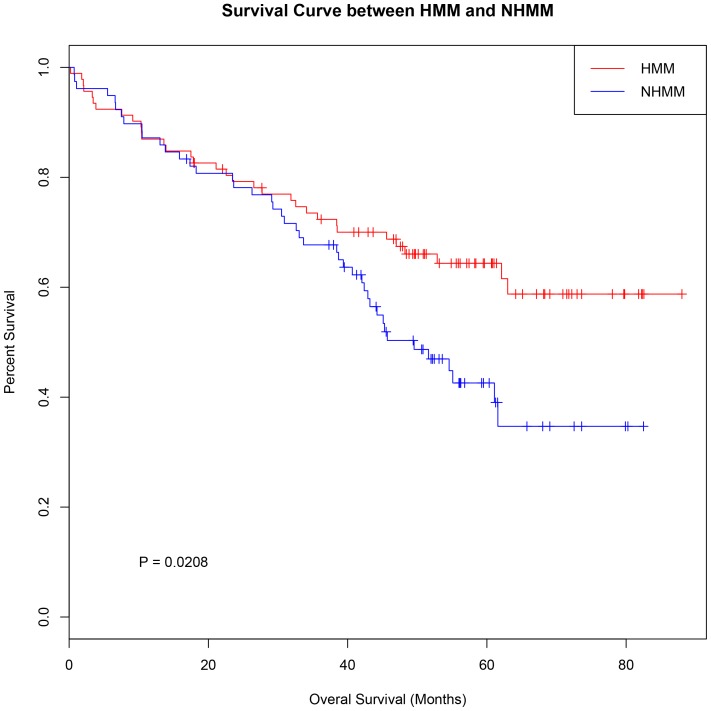
Survival difference between expression-classified HMM and NHMM groups in GSE39754. HMM patients (red) classified by our method have a better overall survival than the NHMM patients (blue). The log-rank test p-value is 0.0208.

The second MM dataset is GSE12896 and GSE39754 with 170 paired gene expression and SNP array profiling samples [10]. The survival outcome of the patients is also available. We used the median copy number of trisomy chromosomes in each sample to call real HMM status of the samples (Figure 8). The expression-classified subtype agreed with copy number-based HMM status at a accuracy of 0.876 (149/170). Furthermore, the expression-classified subtype separated the patients into two groups of significantly different survival (Figure 9, p-value = 0.0208), agreeing with known survival differences between HMM and NHMM groups [4], [8], [9].

### Pool multiple rounds of classification results to improve accuracy

When we applied our classification method trained from GSE6477 to other datasets, we classified each new sample 140 times using the 140 cross-validation models that built from GSE6477. In the above we assigned the final classified subtype using majority voting from the 140 classified subtype calls. We also computed a confidence score for each sample as the proportion of 140 classified subtype calls for the winning majority. When we classified only those samples with confidence score >0.9, the classification accuracy for GSE39754 improved from 0.876 to 0.913 (147/161). This is due to that in the 9 unclassified samples with confidence score <0.9, 7 were misclassified previously. For GSE6401, If we only classified samples whose confidence score is more than 0.9, the accuracy increased from 0.88 (66/75) to 0.93 (62/67).

## Discussion

Multiple myeloma is a malignant cancer of bone marrow plasma cells. Researchers are making progress in diagnosing and treating multiple myeloma in innovative ways; however, it cannot be fully cured by current treatment regimes. The two main aneuploidy-based subtypes of multiple myeloma, HMM and NHMM, correspond to different survival prognosis and potential underlying different pathogenic pathways and altered regulatory networks. Since most expression profiling studies of MM do not measure HMM status, we have developed an expression-based method to classify HMM status of patients with high accuracy across datasets and array platforms. Compared with FISH and copy number based HMM status, the test accuracy in three independent datasets ranges from 0.83 and 0.88. Our method opens up the opportunity for meta-analysis of many MM expression datasets for the disease biology of HMM and NHMM subtypes, and for incorporating HMM status as a covariate in genomics-based survival prognosis models. The lessons we learned in this study, such as using cancer specific characteristics in feature design and pooling multiple rounds of classification results to improve accuracy, are applicable to similar genomics-based classification. We also provide R package and processed datasets publicly at www.ChengLiLab.org/software.

### Use cancer-specific characteristics in classification features

Inferring chromosome abnormalities using gene expression profiles can help predict clinical outcomes and identify causal genomic alterations [14], [20], [21]. In a recent myeloma study, Zhou and colleagues reported cytogenetic abnormalities classification based on gene expression profiles with accuracy up to 0.89 [14]. Similar to their study, our classification method also based on expression data and considered special genes. Due to different purposes, Zhou et al. emphasized copy number-sensitive genes, while we focused on genes in trisomy and non-trisomy chromosomes.

**Figure 10:**
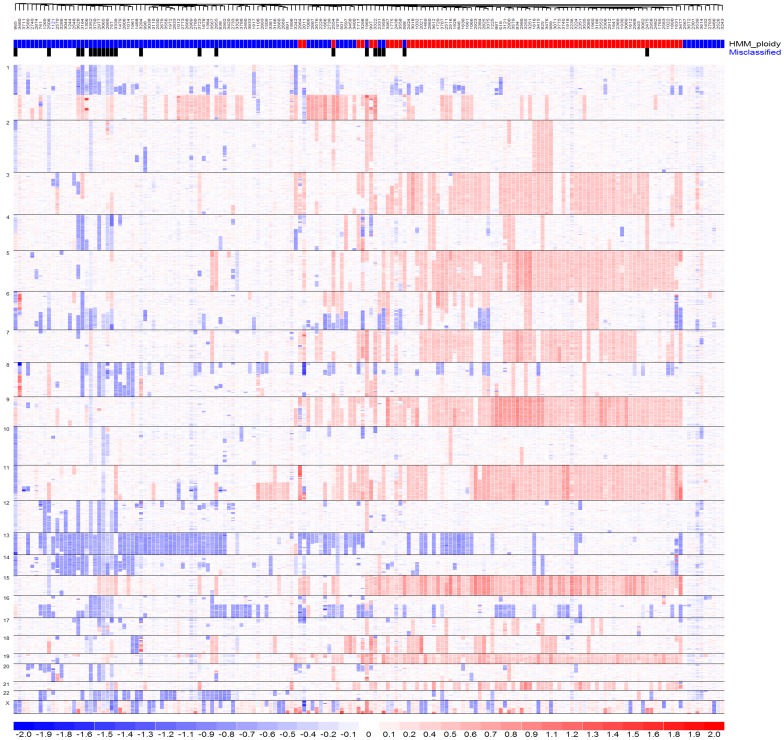
Clustering MM samples in GSE12896 by genome-wide copy numbers. 170 MM samples are clustered from left to right based on their similarities in copy number alterations. SNPs are ordered by chromosome positions from top to bottom. The color scale on the bottom indicates log2 ratios relative to two copies. Blue color indicates copy number loss, white color indicates copy number close to normal, and red color indicates copy number gains. The horizontal red and blue bars on the top indicate sample groups of HMM (red) and NHMM (blue) as determined from Figure 8. Black vertical bars on the top indicate misclassified samples.

Importantly, we found that the carefully selected gene features considering trisomy and non-trisomy chromosomes resulted in more classification power than using the whole list of differentially expressed genes between the two subtypes. We decided that the adjusted TC mean and NTC mean are good features for cross-dataset KNN classification (Figure 5). The classification accuracy is higher than when we use all differential expressed genes as features to classify new samples (Table 1). The reason lies in that we took biological features of multiple myeloma into consideration. Abundant copy number changes of whole and partial chromosomes are a characteristic feature of multiple myeloma and its two subtypes (Figure 10), which lead to dosage effect of many expressed genes located in these chromosome regions. Such dosage alterations affect global gene expression more strongly than platform differences and batch effect do. By using two summarizing features (TC mean and NTC mean) to capture these subtype specific characteristics, such as 8 trisomy chromosomes in HMM and chromosome 13 deletion in NHMM, we achieved better classification accuracy than not considering these characteristics.

**Table 1:** Comparison of accuracy between standard KNN classification using all differentially expressed genes and the two-feature method (k = 16).

Dataset	Standard KNN classification	Two-feature KNN classification
GSE6477	0.85	0.86
GSE19784	0.57	0.83
GSE6401	0.71	0.88

### Causes of misclassified samples and future improvements

**Figure 11:**
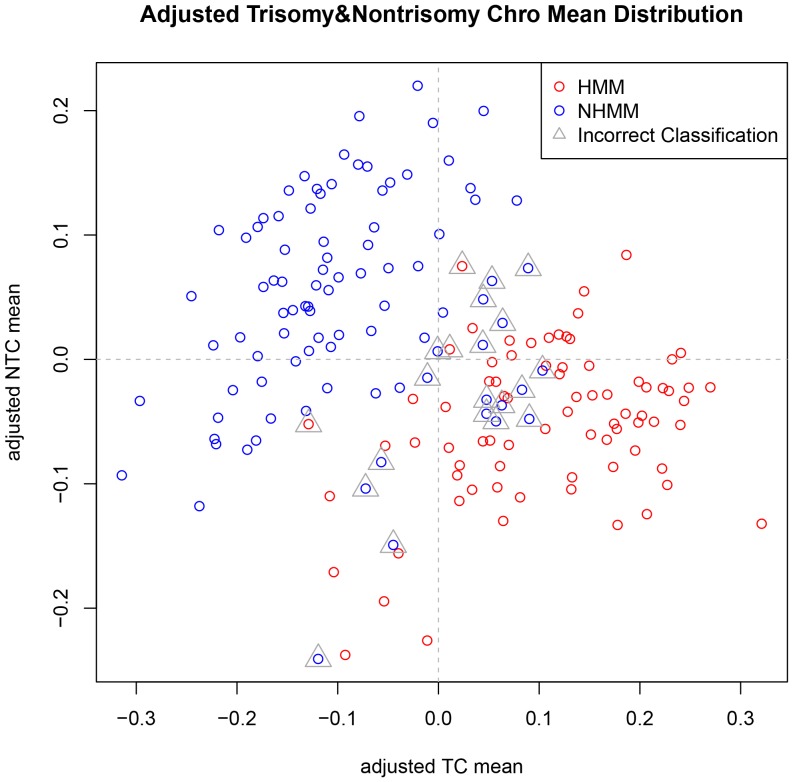
Scatterplot of adjusted trisomy and nontrisomy chromosome means of GSE39754. The green triangles indicate misclassified samples. Most of them locate at the boundary area between the samples of two copy-number based subtypes.

Although we developed an effective method to classify multiple myeloma subtypes, we did not achieve 100% accuracy. There are both biological causes and analytical causes for misclassified samples. Biologically, multiple myeloma genome is not strictly altered following main HMM and NHMM characteristics. Some MM samples could have both trisomy chromosomes and deletion of chromosome 13 (Figure 10, samples in the middle between HMM and NHMM clusters). These samples may possess expression profiles that partially resemble to both HMM and NHMM, and therefore lie in the boundary between the two subtypes in the feature space (such as the samples indicated by triangle in Figure 6 and Figure 11), rendering KNN classifiers unable to call their HMM status with high confidence. We also notice that a portion of misclassified samples in the dataset GSE12896/GSE39754 are NHMM samples with deletion of both chromosome 1 and 4 (Figure 10, misclassified samples on the left, indicated by black vertical bars). These observations point to directions to further improving the classification method based on biological features of interest.

Another biological cause is inaccuracy of FISH-based HMM status, which may probe only a few chromosomes to determine hyperdiploidy. A hypothetical example is a tetraploid sample with 4 copies for most chromosomes. Its transcriptomic profiles may be similar to NHMM due to equal copy numbers of most genes, but FISH could report it as hyperdiploidy.

Analytically, as we used all sample points to adjust the center of a dataset's sample points in the NTC mean vs. TC mean plot (Figure 5), the proportion between real HMM and NHMM samples could affect this adjustment. For example, if a patient cohort contained all HMM samples, we would have classified half of the samples as NHMM since this is the subtype proportion in the training dataset GSE6477. Although HMM proportion is around 55–60% in a typical MM cohort [7], in the 5 datasets we used, the HMM proportion ranges between 39% and 56% as measured by FISH or copy number microarrays (GSE6477: 70/140, 50%; GSE19784: 113/201, 56%; GSE6401: 37/75, 49%; GSE29023: 45/115, 39%; GSE39754: 77/170, 45%). A future improvement in this regard is to iteratively estimate the proportion of HMM samples during classification.

We have also compared to two other classification methods, support vector machine (SVM) and weighted k-nearest neighbors (KKNN). We set GSE6477 as training data and GSE6401 as test data in the same way as the original KNN classification, and k = 16 is used for both KNN and KKNN. SVM classifies only one more sample correctly than KNN (the accuracy increases from 0.88 to 0.89), and KKNN achieves the same accuracy as KNN. Due to potential better performance of KKNN for unbalanced datasets, we removed various number of HMM or NHMM samples from GSE6401 to create unbalanced datasets, and used KKNN to classify HMM status. Table 2 shows that for both KNN and KKNN, the classification accuracy decreases for unbalanced datasets, but KKNN classifies some unbalanced datasets at up to 9% better accuracy than KNN. Therefore the merit of using KKNN for unbalanced datasets is justified in this setting.

**Table 2:** Classification accuracy for original and modified GSE6401 sample sets.

HMM/NHMM	37/23	37/28	37/33	37/38 (original data)	32/38	27/38	22/38	19/38
% of HMM Method	62%	57%	53%	49%	46%	42%	37%	33%
KKNN	0.84	0.87	0.87	0.88	0.86	0.84	0.85	0.80
KNN	0.85	0.86	0.88	0.88	0.86	0.78	0.77	0.71

TC and NTC means are used as features. The kernel ‘optimal’ and ‘rectangular’ of KKNN is used.

Computing a confidence score by pooling results of multiple rounds of classification could improve the accuracy, if samples with low confidence score tend to be misclassified. In our analysis of GSE39754 and GSE6401, not classifying samples with confidence score <0.9 improved classification accuracy from 0.876 to 0.913 and from 0.88 to 0.93, respectively. In the TC mean vs. NTC mean plots (Figure 6 and Figure 11), most samples that were misclassified are located near the boundary between the two subtypes. These samples tend to be classified into either subtype by multiple KNNs and therefore have lower confidence scores. Some misclassified samples do not follow clear HMM or NHMM copy number patterns (Figure 10, samples in the middle, indicated by black vertical bars). Future studies will better define smaller genomics subtypes other than HMM and NHMM, such as those having few chromosomal alterations and those caused by chromothripsis [22].

## Supporting Information

Table S1 **The 675 genes that are intersection gene features among the 140 leave-one-out models of the training dataset GSE6477.** Chro Type: TC: Trisomy chromosomes, NTC: Non-trisomy chromosomes. FDR: the median FDR of all comparisons in the 140 models. HMM.Mean and NHMM.Mean are the mean value of the scaled gene expression in HMM and NHMM samples, respectively.
(XLS)
